# Fingolimod modulates multiple neuroinflammatory markers in a mouse model of Alzheimer’s disease

**DOI:** 10.1038/srep24939

**Published:** 2016-04-27

**Authors:** Nurgul Aytan, Ji-Kyung Choi, Isabel Carreras, Volker Brinkmann, Neil W. Kowall, Bruce G. Jenkins, Alpaslan Dedeoglu

**Affiliations:** 1 Department of Veterans Affairs, VA Boston Healthcare System, Boston, MA 02130, USA; 2 Department of Neurology Boston University School of Medicine, Boston, MA 02118, USA; 3 A. A. Martinos Center for Biomedical Imaging, Department of Radiology, Massachusetts General Hospital and Harvard Medical School, Boston, MA 02114, USA; 4 Department of Biochemistry Boston University School of Medicine, Boston, MA 02118, USA; 5 Development Franchise Neuroscience, Novartis Pharma AGNovartis CampusFabrikstrasse 12CH-4056 Basel, Switzerland

## Abstract

Sphingosine 1-phosphate (SP1) receptors may be attractive targets for modulation of inflammatory processes in neurodegenerative diseases. Recently fingolimod, a functional S1P1 receptor antagonist, was introduced for treatment of multiple sclerosis. We postulated that anti-inflammatory mechanisms of fingolimod might also be protective in Alzheimer’s disease (AD). Therefore, we treated a mouse model of AD, the 5xFAD model, with two doses of fingolimod (1 and 5 mg/kg/day) and measured the response of numerous markers of Aβ pathology as well as inflammatory markers and neurochemistry using biochemical, immunohistochemistry and high resolution magic angle spinning magnetic resonance spectroscopy (MRS). In mice at 3 months of age, we found that fingolimod decreased plaque density as well as soluble plus insoluble Aβ measured by ELISA. Fingolimod also decreased GFAP staining and the number of activated microglia. Taurine has been demonstrated to play a role as an endogenous anti-inflammatory molecule. Taurine levels, measured using MRS, showed a very strong inverse correlation with GFAP levels and ELISA measurements of Aβ, but not with plaque density or activated microglia levels. MRS also showed an effect of fingolimod on glutamate levels. Fingolimod at 1 mg/kg/day provided better neuroprotection than 5 mg/kg/day. Together, these data suggest a potential therapeutic role for fingolimod in AD.

Alzheimer’s disease (AD) is the most common age-related neurodegenerative disease, characterized by progressive memory loss and irreversible cognitive decline.

The extracellular senile plaque deposit of insoluble, aggregated amyloid β (Aβ) peptide is the most prominent neuropathological hallmark of AD^1^. Aβ neurotoxicity is established to be a critical event in AD pathogenesis and correlated with neuronal and synapse loss, which causes synaptic failure resulting in cognitive dysfunction^2^,^3^,^4^. These events are accompanied by a progressive neuroinflammatory reaction involving the activation of microglia and astrocytes around amyloid plaques in AD pathology^5^,^6^,^7^,^8^.

The ultimate role of Aβ plaque-associated microglial inflammatory response remains controversial. Studies have proposed that in the AD brain, microglia are increased around extracellular Aβ plaques, and activate abnormal production of inflammatory mediators that are neurotoxic, suggesting they promote neuronal degeneration in AD^9^,^10^ whereas other studies suggested that microglia play critical roles as mediators of Aβ clearance therefore exerting neuroprotective effects against Aβ toxicity in neurons^11^,^12^.

We previously reported the beneficial effects of nonsteroidal anti-inflammatory drug ibuprofen in lowering the Aβ levels in the triple transgenic mouse model of AD (3xTg-AD)^13^ and in double transgenic Alzheimer mice (APPxPS1)^14^. Prophylactic treatment of 3xTg-AD mice with ibuprofen at 6 months of age showed a significant decrease in intraneuronal oligomeric Aβ and hyperphosphorylated tau immunoreactivity in the hippocampus, and reduced the cognitive decline compared to untreated 3xTg-AD mice^13^. In a double transgenic mouse model of AD (APPxPS1) treatment with ibuprofen provided significant protection against the neuronal markers *N*-acetyl aspartate (NAA) and glutamate detected by magnetic resonance spectroscopy (MRS). In that study, we examined the effects of chronic ibuprofen treatment on amyloid plaque deposition, Aβ peptide levels, and the neurochemical profile measured by MRS. Ibuprofen significantly lowered the plaque burden in the 16- to 18-month-old mice as measured by the percent of cortical area. At this age, we found a decrease in the neuronal markers NAA and glutamate and an increase in the astrocytic markers glutamine and myo-inositol in APPxPS1 mice compared to age-matched non-transgenic mice. Ibuprofen provided significant protection against NAA and glutamate loss, however did not significantly affect myo-inositol or glutamine levels^14^. We similarly found in triple transgenic mice that ibuprofen could protect against NAA and hippocampal loss and that it was protective of myo-inositol increases at early time points in hippocampus, but not at later time points^15^.

Fingolimod is a functional sphingosine 1-phosphate (S1P) receptor antagonist that has been approved for the treatment for MS (Multiple Sclerosis)^16^. It has been shown that the anti-inflammatory effect of fingolimod is mediated through S1P1 receptors on lymphocytes, preventing the migration of these cells from peripheral lymphoid organs into the CNS, suggesting it may protect against neuroinflammation^16^,^17^. Fingolimod can cross the blood–brain-barrier (BBB) therefore showing a potential effect on CNS cells including neurons, astrocytes, microglia, and oligodendrocytes expressing S1P receptors^18^. Recently, it has been reported that treatment with fingolimod is associated with microglial neuroprotective effects, including reduction of pro-inflammatory cytokines and increased levels of brain-derived neurotrophic factor (BDNF)^19^. Doi Y, *et al.*^20^ suggested direct effects of the drug on neurons. However, it remains unclear whether modulation of BDNF by the drug *in vivo* is a direct or indirect effect, and what cell types are involved.

We designed this study to test the effect of fingolimod on amyloid β pathology and neuroinflammation associated with activation of microglia and astrocytes in a mouse model of AD.

## Results

### Effect of fingolimod treatment on Aβ plaque load in 5xFAD mice

**Figure 1:**
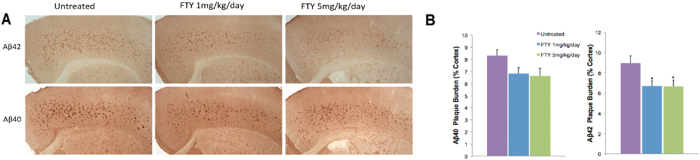
(**A**) Representative pictures showing Aβ40 and Aβ42 immunostained brain sections of 3 months old 5xFAD mice from untreated and 1 mg/kg/day and 5 mg/kg/day of fingolimod treatment groups (Magnification x40). (**B**) Effects of fingolimod treatments on Aβ plaque burden. Brain sections of representative groups were stained for Aβ plaque using Aβ40 and Aβ42 antibodies. 1 and 5 mg/kg/day of fingolimod treatment significantly lowered the Aβ42 plaque burden after 2 months of treatment in the 3 month-old mice compared to the regular diet as measured by the percent of cortical area. Decreased Aβ40 plaque burden was detected in the fingolimod-treated groups however the decreases were not significant. *p < 0.05, (n = 8–10 mice/group). The Aβ40 at 1 mg/kg/day almost reached significance (omnibus ANOVA F = 3.22, p = 0.058).

The 5xFAD transgenic mouse model has been previously reported to exhibit widespread amyloid pathology starting at 2 months old. Here we evaluated the effect of fingolimod treatment at a dose of 1 mg/kg/day and 5 mg/kg/day for 2 months starting at 1 month of age on the deposition of Aβ40 and Aβ42 in the frontal cortex (Fig. 1a). Quantitative analyses of the amyloid beta plaques in frontal cortex shows that both dose of fingolimod treatment resulted in significant decreases in Aβ42 plaque deposition compared with mice given standard drinking water, however the decrease in Aβ40 plaque load did not reach significance with either dose of fingolimod treatment (Fig. 1b).

### Effect of fingolimod treatment on the levels of total Aβ in 5xFAD mice

**Figure 2:**
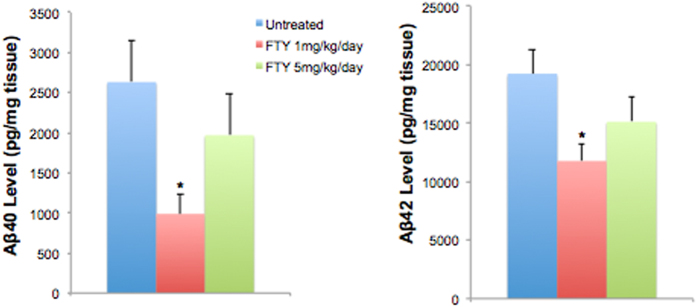
Levels of total (soluble and insoluble) Aβ40 and Aβ42 were quantified by ELISA in the frontal cortex. 1 mg/kg/day of fingolimod treatment significantly decreased the levels of Aβ42 and Aβ40 however 5 mg/kg/day of fingolimod treatment did not reach significant decrease. *p < 0.05, (n = 8–10 mice/group).

We further measured the levels of total Aβ42 and Aβ40 by ELISA in the frontal cortex of 5xFAD mice after 2 months of fingolimod treatment. We found that the levels of total Aβ42 and Aβ40 were statistically lower in 1 mg/kg/day fingolimod-treated group compared to untreated-5xFAD group after 2 months of treatment. However, the levels of total Aβ42 and Aβ40 decrease were not statistically significant in the 5 mg/kg/day treatment group (Fig. 2).

### Effect of fingolimod on intensity of GFAP-positive astrocytes

**Figure 3:**
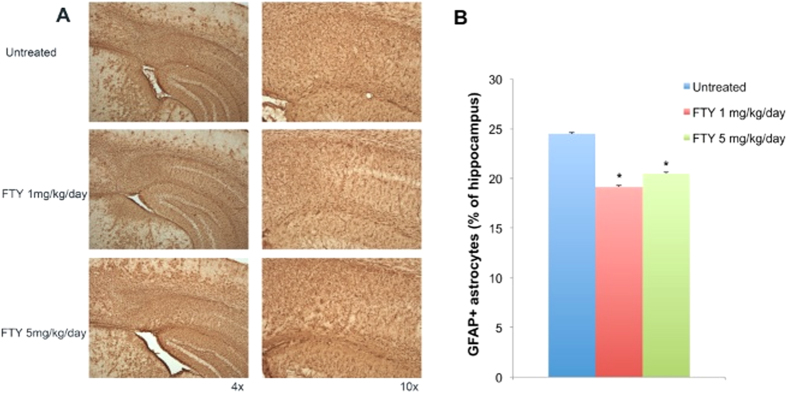
(**A**) Immunohistochemical staining for the astrocytic marker glial fibrillary acidic protein (GFAP) in 5xFAD mouse brain sections from the hippocampus of untreated 5xFAD and fingolimod-treated 5xFAD mouse brains. (**B**) Analysis of GFAP-positive astrocytes in the hippocampus of fingolimod-treated and untreated 5xFAD mice at 3 months of age. 1 and 5 mg/kg/day of fingolimod treatment were significantly decreased the presence of reactive astrocytes in the hippocampus of 5xFAD mice compared to untreated mice. (*p < 0.05), (n = 8–10 mice/group).

Reactive astrocytosis is a well-described pathological process that generally occurs in response to neurodegeneration in AD^21^,^5^. To determine the extent of astrocytosis in the 5xFAD mouse brain, we immunostained brain sections from 3 months old 5xFAD mice and fingolimod-treated groups for GFAP. 5xFAD mice exhibit reactive astrocytosis in several regions of the brain, including the cortex, hippocampus, and striatum that correlated with Aβ plaque load (Fig. 3a).

Densitometric analysis of GFAP immunostained brain sections indicates a significant decrease of astrocytosis in the hippocampus of 1 and 5 mg/kg/day fingolimod-treated mice compared to untreated 5xFAD mice (Fig. 3b).

### Effect of fingolimod treatment on Iba1-positive activated microglia

**Figure 4:**
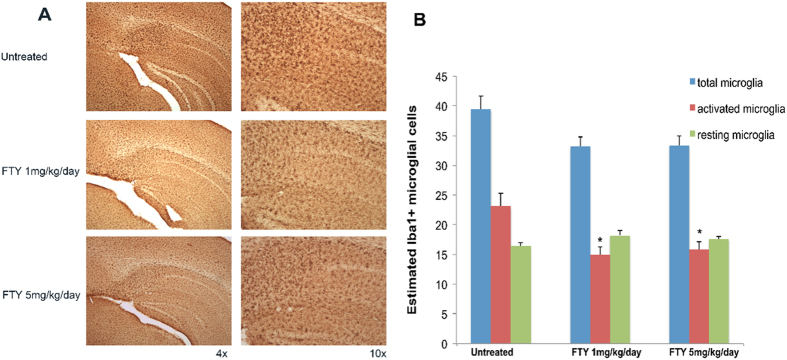
(**A**) Sections of hippocampus and subiculum immunostained for Iba1 in 5xFAD mice untreated and treated with 1 mg/kg/day, and 5 mg/kg/day of fingolimod. Untreated 3 months old 5xFAD mice showed significant increase in the number of activated microglial cells. (**B**) Quantitation of total, active and resting variants of microglia in the hippocampus of 5xFAD untreated and fingolimod-treated groups. The number of activated Iba1-positive microglia significantly decreased in the hippocampus of 1 mg/kg/day, and 5 mg/kg/day fingolimod-treated groups compared with untreated group (*p < 0.05), (n = 8–10 mice/group).

We performed immunostaining for the Iba1 microglial marker at 3 months of age in 5xFAD mice and after fingolimod treatment (Fig. 4a).

Microglia are found in increased numbers in close proximity to Aβ plaques in AD^9^. Microglia associated with Aβ plaques display an activated phenotype characterized by enhanced Iba1-immunoreactivity, retracted processes, perikaryal hypertrophy, and amoeboid appearance that contrast with the microglia not associated with Aβ plaques which display a resting morphology, with small compact somata bearing many long thin ramified processes^5^.

Iba1 immunopositive cell were counted using the optical fractionator method and optical dissector probe. We detected significant differences in the numbers of Iba1-positive activated microglia in the hippocampus of both fingolimod-treated doses in 5xFAD mice group compared to untreated 5xFAD mice.

The number of activated Iba1-positive microglia in the CA1/subiculum region of the hippocampus of fingolimod-treated 5xFAD mice was significantly lower than in the untreated 5xFAD mice group. Moreover, we didn’t observe a significant difference between 1 and 5 mg/kg/day of fingolimod-treated 5xFAD mice group for the number of activated microglia. The number of resting microglia did not show any significant difference between fingolimod-treated and untreated group of 5xFAD mice (Fig. 4b).

**Figure 5:**
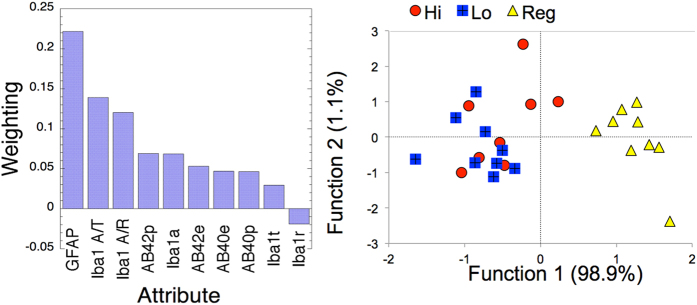
Evaluating the power of the various markers to assess protection with fingolimod. (**A**) Left) Rankings determined from a relief-f algorithm. GFAP has the highest weighting followed by Iba1 quantified by either activated/total (A/T) or activated/resting (A/R), Aβ42 plaque density, Iba1 activated microglia, Aβ42 ELISA, Aβ40 ELISA, Iba1 (total) or Iba1 (resting). (**B**) Right) We then used the top four markers (GFAP, Iba1 (A/T), Aβ42 plaque density and Aβ42 ELISA to perform a linear discriminant analysis (Wilk’s lambda = 0.122; p < 0.0001 for function 1, not significant for function 2). This analysis shows a larger average distance from regular diet for the 1 mg/kg/day fingolimod treatment (Lo) than for the 5 mg/kg/day dose (Hi).

We also evaluated the rankings of the various measures we made for classification of the groups. We used a relief-f attribute selector^22^ to rank the various attributes with regards to classification. The results (Fig. 5a) showed that the GFAP contributed the largest weights, followed by Iba1 (activated/total) or Iba1 (activated/resting), and Aβ42 plaque density. We then used the top four attributes (GFAP, Iba1 (activated/total), Aβ42 plaque density, Aβ42 ELISA) to perform cluster analysis using a simple k-means. This analysis showed that there were only two clusters of which 9/9 regular diet mice were in one cluster with 1 each of the 1 and 5 mg/kg/day and the other 15 fingolimod-treated mice were clustered together. We also performed linear discriminant analysis (LDA), as well as support vector machines (SVM) using a logistic function to demonstrate the separability of the groups. The SVM and the LDA showed similar efficacy with 100% accurate separation of the regular diet from the fingolimod-treated animals and mixing of the two dose groups. However as shown in Fig. 5b, the overall distance of the 5 mg/kg/day group was closer to the regular diet than was the 1mg/kg/day group. The confusion matrix showed mixing between the high and low dose groups. For SVM 9/9 regular diet were classified as regular diet, 5/9 low dose diet were classified as low dose diet and 4/9 were classified as high dose diet, and 1/8 high dose diet was classified as regular dose diet, 5/8 were classified as low dose diet and 2/8 were classified as high dose diet. For LDA 9/9 regular diet were classified as regular diet, 6/9 low dose diet were classified as low dose diet and 3/9 were classified as high dose diet, and 0/8 high dose diet was classified as regular dose diet, 4/8 were classified as low dose diet and 4/8 were classified as high dose diet.

### Magnetic resonance spectroscopy of changes in brain neurochemicals

**Figure 6:**
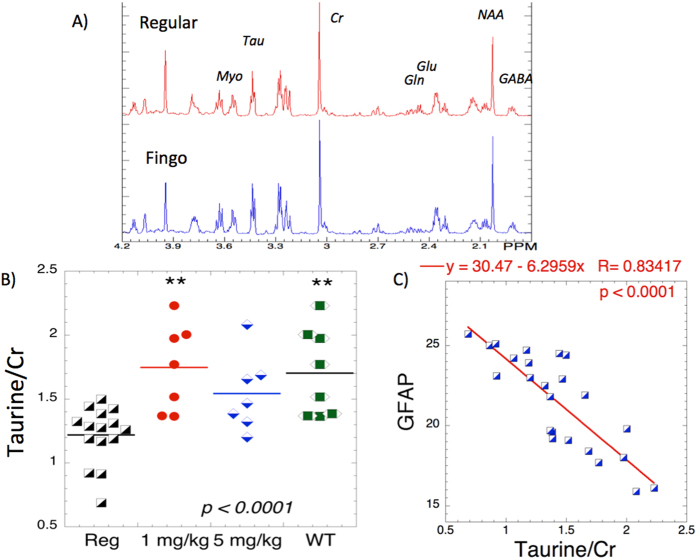
HRMAS of fingolimod treatment in 3 months old mice. (**A**) Representative HRMAS spectra from hippocampus in 5xFAD normal diet and fingolimod-treated (1 mg/kg/day) a few molecules including N-acetylaspartate (NAA), glutamate, glutamine, creatine, taurine and myo-inositol are labeled. (**B**) Effect of fingolimod treatment as a function of dose on taurine levels. There is a very significant effect on raising taurine levels in the fingolimod-treated mice. (**C**) Correlation between taurine levels and GFAP staining in the mice that had both measured. There is a very strong correlation R = 0.83; p < 0.0001.

Two MRS studies of 5xFAD mice have appeared, one from our lab^23^ and one from another lab Mlynarik *et al.*^24^. The changes observed in both studies were very similar (decreased NAA, glutamate and GABA, and increased glutamine and myo-inositol). Our data showed large numbers of neurochemical changes that could be ameliorated using treatment with scyllo-inositol and the NSAID flurbiprofen. That study was conducted on mice 8 months of age. The mice studied here were three months of age. At three months of age the neurochemical changes are considerably less profound than at 8 months of age. We compared four groups of mice (WT, 5xFAD regular diet and 1 mg/kg/day and 5 mg/kg/day of fingolimod). We found trends towards decreased NAA, GABA and glutamate at three months that was barely significant for glutamate (p < 0.05; F_3,36_ = 3.1) and was protected by fingolimod. There was a large decrease in taurine that was highly significant and was protected by fingolimod (p < 0.001; F_3,36_ = 7.3). These data are shown in Fig. 6. We found an unusually strong inverse correlation between GFAP staining and taurine levels in the hippocampus of the mice (R = 0.84; p < 0.0001). We also performed similar correlations between Iba1 staining and as well as ELISA measures of Aβ40 and Aβ42 and Aβ40/42 plaque density. The correlation between taurine and Iba1 activated was not very good (R = 0.3), while the correlations with Aβ40/42 plaque density were better (R = 0.47; 0.41 respectively) and better still for Aβ40/42 ELISA (R = 0.65, 0.67 respectively; p < 0.001 for both). These data are of interest since taurine has been shown to act as an endogenous anti-inflammatory chemical^25^.

## Discussion

Fingolimod displays potent anti-inflammatory effects in MS patients and animal models of neurodegenerative disease. Therefore we investigated the effect of fingolimod treatment on neuroinflammation, amyloid pathology and neurochemical markers in a transgenic mouse model of AD.

Our results indicate that fingolimod intake reduced the levels of Aβ and plaque burden in 5xFAD mice and correlated with the significant decrease of activated microglia measured using Iba1 and the astrocytic inflammatory marker GFAP.

Fingolimod treatment has been previously reported to improve cognitive function in a rodent model of AD. Chronic administration of fingolimod (1 mg/kg, i.p., 14 days) significantly attenuated the Aβ42-induced learning and memory impairment and prevented the hippocampus neuronal damage in a rat model of AD^26^. Consistent with these results, it has been found that treatment with fingolimod protects against Aβ1-42-induced cognitive impairment in a mouse model of AD^19^.

Further, neuroprotective effects of fingolimod treatment have also been reported in Aβ1-42 induced neurotoxicity *in vitro* in cultured mouse primary cortical neurons^20^.

On the other hand, 6-days of treatment of APP transgenic mice with fingolimod (0.5 mg/kg/day) has been reported to decrease the levels of soluble Aβ40 and, interestingly, increase the levels of soluble Aβ42^27^. These results were not consistent with observations from their *in vivo* results, in which fingolimod treatment was shown to decrease the levels of Aβ in cultured neuronal cells. They considered that fingolimod might have a distinct effect on Aβ42 metabolism through altering the clearance of Aβ42 by microglial cells *in vivo*.

Recently, the neuroprotective effect of fingolimod has been shown in cerebral white matter injury following neonatal hyperoxia^28^. In a neonatal model of hyperoxia, fingolimod (1 mg/kg) treatment reduced hyperoxia-induced cognitive dysfunctions, microglia activation and associated pro-inflammatory cytokine expression^28^.

Although the neuropathological mechanisms between AD and white matter damage remain unclear, white matter ischemic damage is considered to be an important contributor to the pathology and development of dementia in AD. Ischemia has also been revealed to increase the production of Aβ and facilitate cognitive impairment in animal models^29^,^30^.

The neuroprotective effect of fingolimod on human astrocytes, has been shown through the induction of neurotrophic factors and inhibition of inflammatory genes^31^ suggesting, a part of the effects of fingolimod might be mediated via astrocytes.

Although many experiments strongly suggest a role for microglia in Aβ42 phagocytosis or clearance, ongoing controversy exists regarding whether activated microglia contribute to their maintenance or clearance.

Our results demonstrate that administration of 1 mg/kg/day of fingolimod for 2 months results in a significant decrease in total Aβ42 plaque burden and the levels of Aβ40 and 42 in the brains of 3 months old 5xFAD mice and that lower-dose (1 mg/kg/day) of fingolimod treatment was more effective in decreasing Aβ40 and 42 levels compared to treatment with the higher-dose (5 mg/kg/day). However, treatment with either low or the high dose resulted in significant decrease in Aβ40 plaques. Effect of the low and the high dose on Aβ40 and 42 plaques, activated microglia and astrocytes was comparable. Lower dose was also more effective in increasing taurine levels than the high dose.

In addition to its effects on lymphocyte egress from lymph nodes, fingolimod has been also shown to modulate a variety of intracellular signaling pathways in different cell types following the internalization of S1P1 receptors suggesting, this compound has a more complex mechanism^32^,^33^,^34^.

The fact that lower dose work better than higher dose may indicate that complete removal of S1P1 by fingolimod (through internalization and degradation) may not be the one and only beneficial effect. It could be that some agonistic effects/signaling by drug in one or the other cell type/S1PR could also contribute, and this occurs more at the lower dose when internalization is incomplete.

The results from fingolimod phosphate (FTY720P)-mediated signal transduction experiment in primary human umbilical vein endothelial cells shows that despite S1P1 receptors complete internalization induced by a transient exposure to FTY720P, S1P1 receptors were able to maintain the transmission of the persistent signaling activity from receptor-proximal G proteins to downstream effector systems^34^.

These results suggest that FTY720P remains bound to internalized S1P1 receptors and maintains an active conformation that targets the receptor for atypical signaling, processing and trafficking.

In addition, S1P1 has been shown to interact with the regulator of G protein signaling (RGS)-2 protein, which is a GTPase-activating protein (GAP), to regulate cell motility in a concentration dependent manner. S1P1 responds to S1P ligand stimulation with a typical bell-shaped dose curve, and the RGS2–S1P1 complex dissociated at higher S1P concentrations in S1P1-expressing CHO cells. Similar to these results, they observed that FTYP-induced cell motility followed a similar bell-shaped dose–response curve and cell mobility was also decreased at higher concentrations of FTYP. The S1P1–RGS2 complex was stable upon stimulation with lower FTYP concentrations, whereas the complex dissociated at higher concentrations. These results suggest that the decreased S1P1-mediated cell motility at high doses of FTYP might be through a mechanism acts independently of S1P1 internalization^32^.

At low dose (10 nM), chronic fingolimod treatment (72 h) has been shown to stimulates the differentiation of oligodendrocyte precursor cells (OPCs) and expression of myelin basic protein (MBP)-positive cells compare to higher dose (1μM) of fingolimod treatment in cultured rat OPCs, indicating that the drug may have a dose and time dependent effect on developing oligodendrocytes^33^.

Based on the literature, our results support the hypothesis that the effect of fingolimod on both Aβ42 and inflammation with different doses could be mediated via different signaling mechanisms through S1P receptors in neuron and glial cells.

In conclusion, these studies showing the beneficial effect of fingolimod treatment in different cell types represent that a dose and time dependent treatment of fingolimod could reflect a distinct property of this agonist/receptor pair resulting in a sustained activation of receptor internalization and active signaling following exposure to fingolimod.

**Figure 7:**
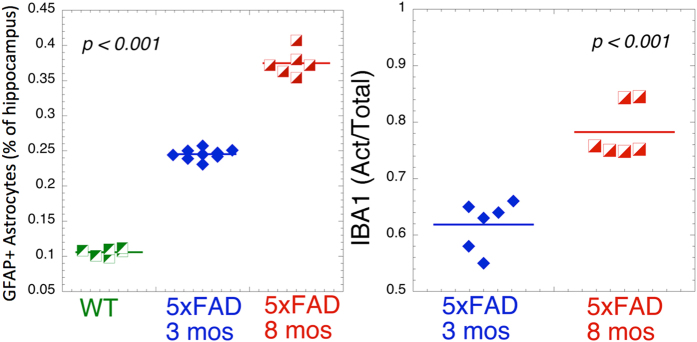
Effects of age on inflammatory markers in hippocampus in 5xFAD mice. Left) GFAP staining shows a large increase from WT to 3 months and then 8 months old mice (P < 0.0001 for differences between all groups using ANOVA with a Tukey-HSD post-hoc test). Right) Increased activated/total microglia ratio for 3 and 8 months old animals (p < 0.0001 for difference between groups. There were no activated microglia noted in the WT mice.

In contrast to our previous study of 5xFAD mice at 8 months of age^23^ the mice in this study were only three months of age. Although there is already some early pathology in these mice at this age (for instance increase Aβ42 plaque deposition)^35^ there is a dramatic increase in many pathological markers from 3 months to 8 months including an increase in Iba1 staining (ratio of activated/total microglia) from 0.58 ± 0.07 at 3 months to 0.78 ± .043 at 8 months (unpaired t-test, t value −6.19, p < 0.0001) and in GFAP staining from 0.106 ± 0.005 in WT to 0.245 ± 0.007 at 3 months to 0.375 ± 0.018 at 8 months of age (F_2,20_ = 867.33, p < 0.0001; Tukey post-hoc tests show all three groups are significantly different from each other at p < 0.0001) (see Fig. 7). In line with this age dependence, we found a total decrease in the number of large neurochemical changes in the hippocampus at 3 months of age compare to 8 months of age that we reported earlier^23^. At three months of age the largest change was in taurine levels (see Fig. 6). Even more striking, there was a very strong correlation between taurine levels and GFAP staining (Fig. 6). These data suggest that fingolimod increases taurine levels and that this may be one mechanism behind the neuroprotection noted. Taurine is the amino acid with the highest concentration in the body and has numerous different roles including osmoregulation as well as acting as an indirect anti-oxidant and endogenous anti-inflammatory agent^36^,^37^,^25^. Given that cells exposed to high oxidative stress also show high taurine levels, it is logical to assume that the inverse correlation between GFAP staining and taurine levels may reflect protective effects of taurine. Oral supplementation with taurine has shown some success for neuroprotection in models of traumatic brain injury^38^. Whether fingolimod increases taurine levels, or alters the cellular distribution of taurine containing cells is a topic for further investigation. Since rodents have much higher concentrations of taurine than do humans^39^, it would be of interest to see if such relations also hold for human tissue between brain taurine levels and neuroinflammatory markers. The fact that we observed strong correlations with taurine for GFAP as well as ELISA measurements of Aβ40/42, but not with activated microglia or with plaque density measures suggests that taurine is more closely associated with astrocytic activity rather than activated microglia. Since taurine is readily measureable using MRS in both humans and mice, it may provide a non-invasive marker for modulation of neuroinflammation in the brain.

## Material and Methods

### Mice

Female 5xFAD transgenic mice^35^ and non-transgenic female littermates as controls were used in this study. 5xFAD mice coexpress the human APP and PS1 genes harboring a total of 5 FAD mutations [APP K670N/M671L (Swedish) + I716V (Florida) + V717I (London) and PS1 M146L + L286V] under the control of the murine Thy-1 promoter. The study comprised 4 groups of mice: transgenic mice treated at 2 different doses of fingolimod, untreated 5xFAD mice, and untreated non-transgenic (WT) mice (n = 10). Mice were housed on a 12 h light:12 h dark schedule. All mice were given access to food and water ad libitum. All animal experiments were carried in accordance with the NIH Guide for the Care and Use of Laboratory Animals and were approved by research animal care committee at VA Boston Healthcare System. The Medical Center is fully AAALAC accredited as an approved research facility and the hospital has on file an Assurance of Compliance with Public Health Service regulations and requirements and provisions of the Animal Welfare Act.

### Diet and drug protocol

Fingolimod was kindly provided by Volker Brinkmann, Novartis Pharma AG Basel, Switzerland. Fingolimod, added into the drinking tap water, was tested at two different doses, 1 mg/kg/day and 5 mg/kg/day. Water was changed twice weekly. Food and water consumption were monitored weekly. We monitored mice for general well being, and no side effects related to either treatment were observed.

### Tissue collection

At 3 months of age, mice were euthanized and brains removed for analysis. The left hemisphere was post-fixed with the 4% paraformaldehyde solution for 24 h and cryoprotected in a graded series of 10% and 20% glycerol/2% DMSO solution for histological analysis. The right hemisphere was dissected at the frontal level and saved for the Enzyme-linked immunosorbent assay (ELISA) assay. Tissue punches of freshly frozen hippocampus were collected for MRS studies.

### Histology/Immunohistochemistry

Brains were serially cut at 50 μm on a freezing microtome. Immunohistochemical procedures were performed as previously described^40^. In brief, free-floating sections were incubated overnight in primary antibody followed by PBS (Phosphate buffered saline) washes and incubation in peroxidase-conjugated secondary antibody followed by development using 3,3′-diaminobenzidine tetrahydrochloride (DAB) as a chromagen. The antibodies used were Aβ1–40 and Aβ1–42 (Invitrogen, Grand Island, NY) to define Aβ deposits, ionized calcium binding adaptor molecule 1(Iba1) (Wako Chemicals, USA) to stain microglia, and glial fibrillary acidic protein (GFAP) (Chemicon, USA) to stain astrocytes.

### Quantitative analysis of Aβ deposits and intensity of GFAP-positive astrocytes

For the estimation of cortical Aβ plaque burden, three serial sections per mouse brain were analyzed blindly using a custom software written in Matlab. The most rostral section analyzed was at the anterior commissure level (~0.1 mm anterior to bregma), and each successive section was at 0.34 mm increments. Images of the Aβ stained sections were acquired using a Nikon Optiphot microscope at 4x magnification. The cortical region of interest was processed in Photoshop together with an area from the same image that did not contain plaques in order to control for background stain. Using both images in the Matlab program we determined the percent plaque area.

Similarly, the percentage of GFAP-positive astrocytes was analyzed using the Mat Lab program in the subiculum/CA1 region of the hippocampus in 3 sections per mice.

### Quantitative analysis of Iba1-positive microglia cell number

To quantify microglia, we applied the Optical Fractionator probe in the StereoInvestigator software (MBF Bioscience, Williston, VT) using two different markers to count resting microglia and activated microglia based on their morphological differences. Tree sections from the hippocampus between bregma levels −2.5–3.0 mm were selected for each mouse. The region of interest, the subiculum/CA1 area of the hippocampus was delineated at 4x magnification. Cell counting was performed at 40x magnification in a counting frame of 350 × 350 μm.

### ELISA assay

Dissected frontal brain tissue was homogenized with 8 ×  5 M guanidine HCl buffer to analyze the level of total (soluble + insoluble) Aβ. To determine Aβ levels, human Aβ40 and Aβ42 ELISA kits (Invitrogen, Grand Island, NY) were used according to manufacturer’s specifications. Briefly, homogenized samples were added into the wells of a 96-well plate, samples were then mixed with a cleavage-specific antibody to either Aβ40 or Aβ42. After overnight incubation at 4 °C, plates were washed and incubated with the secondary antibody for 30 minutes at 25 °C. Washed wells were developed by the addition of a substrate. The substrate reaction was then stopped and color intensity was measured at 450 nm.

### High resolution magic angle spinning spectroscopy (HRMAS)

*In vitro* MRS was collected as previously published^41^,^14^. We collected high resolution magic angle spinning (HRMAS) spectra on Bruker 14T (Billerica, MA). We obtained tissue punches of freshly frozen hippocampus from mice. The punches were 1 mm in diameter and were taken from hippocampus. The dissected tissue sample was placed into a glass cylinder positioned in a 3 mm zirconium oxide MAS rotor (volume 50 μL). HRMAS measurements were performed using a sample spinning rate of 3.6 kHz selected to push the spinning side bands outside the frequency region of the metabolites. The experiments were performed at 4 °C to minimize tissue degradation.

Data were acquired using a rotor synchronized, T_2_-filtered Carr–Purcell–Meiboom–Gill (CPMG) pulse sequence [90 − (τ − 180 − τ − Acq)_n_] with two different effective TEs (100 ms/10 ms). The longer TE serves to remove the lipid/macromolecular resonances and the short TE retains them. The interpulse delay, τ, is synchronized to the rotor frequency, and is 272μs. The n value for the relatively short T_2_ filter was 36 and for the long TE was 360. The short τ value removes all the T_2_^*^ - like effects on the lineshapes. The long T_2_ filter yields approximately 95% of the total spectral intensity of all metabolites of interest compared to the short TE. Other acquisition parameters were a 90° pulse of 5–10 μs, a spectral width of 8 kHz, 16 K complex points, 256 averages and a TR of 5 s. Samples were placed in the rotor with a small amount of D_2_O (Sigma-Aldrich, Milwaukee, WI) for locking and shimming.

Data were analyzed using the Chenomx (Edmonton, Alberta, Canada) package fitting the entire metabolite spectrum for each neurochemical. HRMAS data were reported as molar ratios to creatine since our prior studies of the absolute concentrations in multiple different AD transgenic mouse models showed no change in total creatine between WT and any of the AD models^41^,^14^,^42^. In a paper recently published by Mlynarik *et al.*^24^ examining the 5xFAD mice using a water normalization method, no significant change in the creatine concentrations was noted in the 5xFAD mice compared to WT. Statistical analyses of the MRS data were performed using a one-way ANOVA with Tukey HSD post-hoc tests for between group comparisons for each metabolite. Classification of the data was performed using Weka^43^.

### Statistical analyses

Statistical analyses of the data were performed using a one-way ANOVA with Tukey HSD post-hoc tests for between fingolimod-treated and untreated groups.

## Additional Information

**How to cite this article**: Aytan, N. *et al.* Fingolimod modulates multiple neuroinflammatory markers in a mouse model of Alzheimer’s disease. *Sci. Rep.* **6**, 24939; doi: 10.1038/srep24939 (2016).
